# Supplementary material for: Relationship between Health Status and Daily Activities Based on Housing Type among Suburban Residents during COVID-19 Self-Isolation
Source: Int J Environ Res Public Health. 2023 Feb 1;20(3):2639. doi: 10.3390/ijerph20032639 (PMC9914989; doi:10.3390/ijerph20032639)
Supplement: Supplementary file 1 [file ijerph-20-02639-s001.zip › ijerph-2124491-supplementary.pdf]

Supplementary material

A. Let me ask you about yourself.

A1 Please indicate your gender and age. (circle one for each).

【Gender】 1．Male 2．Female

【Age】 1．20～29 2．30～39 3．40～49 4．50～59
5．60～64 6．65～69 7．70～79 8．Over 80

A2 Please answer the following questions about your occupation. (circle one)

1．Company employees and civil servants
2．Self-employed profession
3．Agriculture and forestry
4．Household chores
5．Students
6．Part-time job
7．Fixed year retirees
8．Other（ ）

A3 Please indicate in the answer the neighborhood association where you live.

Teraichidai, Tondabayashi City [chome]

A5 Please describe your family structure. (circle one)

1．Living alone
2．Couple
3．Nuclear family households
4．Three-generation households
5．Other（ ）

A6 Please answer the following questions about your residence. (circle one)

1．Detached house
2．Apartment

B We would like to ask you a few questions about your daily activities.

*Looking back over the past week, please answer the following questions about your typical day.

B1 How long do you perform the activities of daily living? (circle one for each)

		I don't	Less than 1 hour	1〜3 hours	4〜6 hours	Over 6 hours
1	Patrolling					
2	Meals					
3	Commuting to work and school					
4	Work					
5	Study					
6	Housework					
7	Care and nursing					
8	Childcare					
9	Shopping					
10	Moving					
11	Television					
12	Rest					
13	Personal development					
14	Recreational hobbies					
15	Sports					
16	Social participation activities					
17	Exchange socializing					
18	Medical treatment					
19	Sleep					

C We would like to ask you about your health.

*We ask about your daily health over the past week. Please answer the questions with your first impression. (circle one for each)

C1． In general, would you say your health is？

0	1	2	3	4
Poor	Fair	Good	Very good	Excellent

C2． Moderate activities, such as moving a table, pushing a vacuum cleaner, bowling, or playing golf？

1	2	3
Yes, limited a lot	Yes, limited a little	No, not limit at all

C3． Climbing several flights of stairs？

1	2	3
Yes, limited a lot	Yes, limited a little	No, not limit at all

C4． Accomplished less than you would like？

0	1	2	3	4
None of the time	A little of the time	Some of the time	Most of the time	All of the time

C5． Were limited in the kind of work or other activities？

0	1	2	3	4
None of the time	A little of the time	Some of the time	Most of the time	All of the time

C6． Accomplished less than you would like？

0	1	2	3	4
None of the time	A little of the time	Some of the time	Most of the time	All of the time

C7． Did work or other activities less carefully than usual？

0	1	2	3	4
None of the time	A little of the time	Some of the time	Most of the time	All of the time

C8. During the past 4 weeks , how much did pain interfere with your normal work (including both work outside the home and housework)?？

0	1	2	3	4
Not at all	A little bit	Moderately	Quite a bit	Extremely

C9. Have you felt calm and peaceful?？

0	1	2	3	4
None of the time	A little of the time	Some of the time	Most of the time	All of the time

C10. Did you have a lot of energy？

0	1	2	3	4
None of the time	A little of the time	Some of the time	Most of the time	All of the time

C11. Have you felt downhearted and depressed？

0	1	2	3	4
None of the time	A little of the time	Some of the time	Most of the time	All of the time

C12. During the past 4 weeks , how much of the time has your physical health or emotional problems interfered with your social activities (like visiting friends, relatives, etc.)？

0	1	2	3	4
None of the time	A little of the time	Some of the time	Most of the time	All of the time
